# The Stability, Sustained Release and Cellular Antioxidant Activity of Curcumin Nanoliposomes

**DOI:** 10.3390/molecules200814293

**Published:** 2015-08-05

**Authors:** Xing Chen, Li-Qiang Zou, Jing Niu, Wei Liu, Sheng-Feng Peng, Cheng-Mei Liu

**Affiliations:** State Key Laboratory of Food Science and Technology, Nanchang University, No. 235 Nanjing East Road, Nanchang 330047, China; E-Mails: ncuskchenxing@163.com (X.C.); zouliqiang2010@163.com (L.-Q.Z.); niujing28@sina.com (J.N.); psf01416@163.com (S.-F.P.); chengmeiliu@yahoo.com.cn (C.-M.L.)

**Keywords:** curcumin nanoliposomes, stability, sustained release, cellular antioxidant activity, cellular uptake

## Abstract

Curcumin is a multifunctional and natural agent considered to be pharmacologically safe. However, its application in the food and medical industry is greatly limited by its poor water solubility, physicochemical instability and inadequate bioavailability. Nanoliposome encapsulation could significantly enhance the solubility and stability of curcumin. Curcumin nanoliposomes exhibited good physicochemical properties (entrapment efficiency = 57.1, particle size = 68.1 nm, polydispersity index = 0.246, and zeta potential = −3.16 mV). Compared with free curcumin, curcumin nanoliposomes exhibited good stability against alkaline pH and metal ions as well as good storage stability at 4 °C. Curcumin nanoliposomes also showed good sustained release properties. Compared with free curcumin, curcumin nanoliposomes presented an equal cellular antioxidant activity, which is mainly attributed to its lower cellular uptake as detected by fluorescence microscopy and flow cytometry. This study provide theoretical and practical guides for the further application of curcumin nanoliposomes.

## 1. Introduction

Curcumin, a yellow colored, low molecular weight polyphenol, is extracted from the rhizome of *Curcuma longa* [1]. This natural polyphenol has been used to treat a variety of inflammatory and other diseases for centuries, and has attracted considerable attention due to its nontoxicity, even at high doses (12 g/day in humans) [2] and multiple pharmacological activities, namely anti-inflammatory, antioxidant, antiviral, antibacterial, antifungal as well as antitumor effects [3,4]. Despite its potential health benefits to humans, the poor water solubility, instability in *in vivo* and *in vitro* environments and the extremely low bioavailability of curcumin greatly limit its applications in the food and pharmaceuticals industries [5]. Only a trace amount of curcumin appears in blood plasma, even after high dose intake, and orally administered curcumin is most excreted with the faeces and urine after rapid metabolization to form several reduced products in the intestine [6].

Many attempts to overcome these limitations of curcumin through various encapsulation methods such as microcapsules [7], micelles [8], complexes [9], emulsions [10] and liposomes [11,12,13,14] have been reported. The utilization of encapsulations can protect the core material from adverse environmental conditions, and improve the half-life of compounds in both *in vivo* and *in vitro* systems, and thus enhance their bioavailability [15]. Among these encapsulation methods, nanoliposomes are a potentially good delivery system due to their nanometer level size, biodegradability, low toxicity and capacity to encapsulate both hydrophobic and hydrophilic compounds [16].

Curcumin easily degrades when exposed to alkaline conditions [17], and when chelated with metal ions [18], thereby resulting in instability. Matloob *et al.* [19] reported that the solubility and stability of curcumin in fetal bovine serum could be improved by liposomal incorporation. Liposomes could improve the water solubility, neutral and thermal stability of curcumin [14]. To our knowledge, there are no reports about whether curcumin could be stabilized by nanoliposomes when exposed to adverse alkaline solution and metal ion solution conditions. Therefore, the focus of the present work was whether the stability of curcumin could be improved by nanoliposomes when exposed to adverse alkaline solutions and metal ion solutions.

It has been reported that liposomal curcumin showed higher stability and DPPH scavenging activity than free curcumin, which is based on a simple chemical reaction [6]. Biological systems are much more complex than simple chemical mixtures, and science to evaluate their antioxidant activities solely by chemical antioxidant assays is lacking. The cellular antioxidant activity (CAA) assay is a cell-based method which takes some factors into account, such as cell uptake, metabolism, and distribution of bioactive compounds, thus CAA may better predict antioxidant behavior in biological systems. It is necessary to further evaluate the antioxidant activity of curcumin after liposomal encapsulation through the CAA method. The cellular uptake capacity of curcumin liposomes was measured to explain the CAA results.

In this study, curcumin nanoliposomes were prepared by combining thin film and dynamic high-pressure microfluidization (DHPM). The physicochemical characterization of the resulting curcumin nanoliposomes, including morphology, particle size, polydispersity index, zeta potential and drug entrapment efficiency is described in details. The sustained release properties of the curcumin nanoliposomes were investigated. The stability of curcumin liposomes against different pH buffers and metal ions solution was explored. The cellular antioxidant activities and cellular uptake capacity of curcumin nanoliposomes were evaluated.

## 2. Results and Discussion

### 2.1. Physicochemical Characterization and Morphology of Curcumin Nanoliposomes

**Figure 1:**
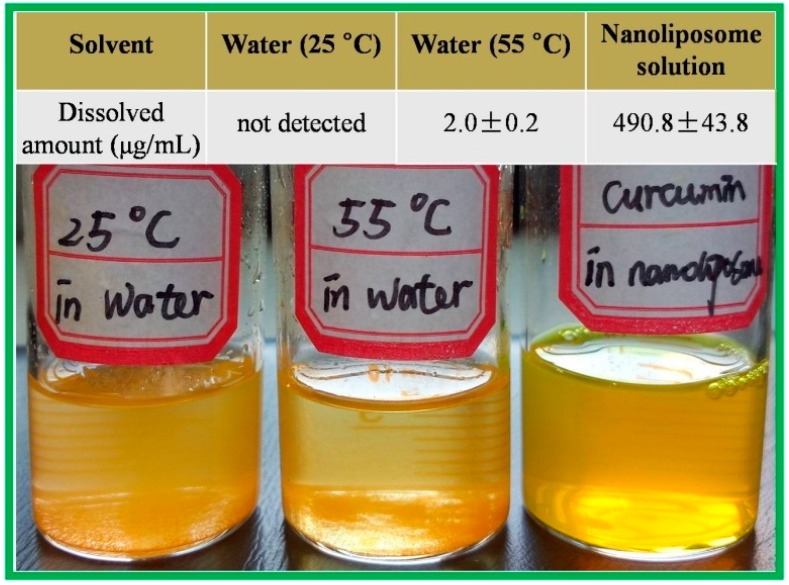
Curcumin dispersed in three solvents.

As shown in Figure 1, the curcumin nanoliposomes formed a stable and dispersed system, but there were a lot of curcumin crystals in the mixtures of curcumin and water incubated at both 25 °C and 55 °C. The high solubility of curcumin in nanoliposomes (490.8 μg/mL) indicated that the water solubility of curcumin was significantly improved by nanoliposome encapsulation.

**Figure 2:**
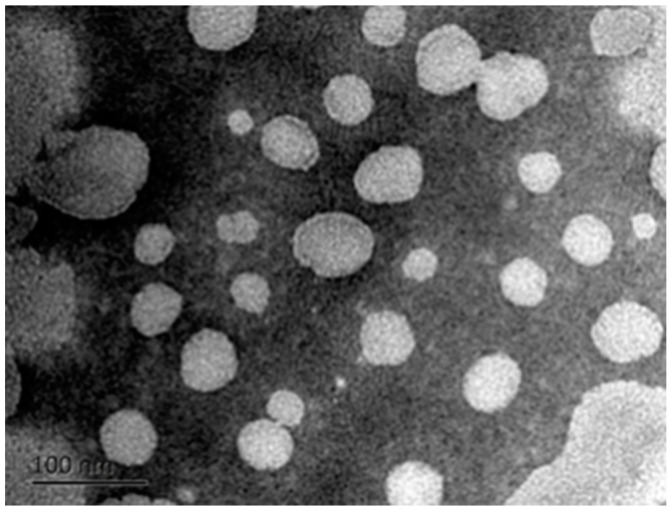
TEM micrograph of curcumin nanoliposomes.

The encapsulation efficiency of the curcumin nanoliposomes was 57.1% ± 1.1%, which is within the range of previous results (from 41% to 82.3%) [6,12,13,14]. The zeta potential of our curcumin nanoliposomes was about −3.16 ± 0.34 mV, which is similar to the results of Saengkrit *et al.* [20], but much lower than the results of Liu *et al.* [14] (−24.37 ± 4.63 mV), Chu *et al.* [21] (−22.6 ± 0.88 mV) and Niu *et al.* [6] (−48 mV). Liposomes are mainly composed of cholesterol and phospholipids. The difference in zeta potential can probably be attributed to variations in the competence and properties of the phospholipids. The average diameter and polydispersity index of the curcumin nanoliposomes were 68.1 ± 1.5 nm and 0.246, respectively. This was mainly attributed to the DHPM treatment which uses strong mechanical forces and could significantly reduce the particle size of liposomes [22,23,24]. As shown in Figure 2, the apparent morphology of the curcumin nanoliposomes was one of spherical defined shapes, and the particle size was consistent with the results obtained by DLS.

**Figure 3:**
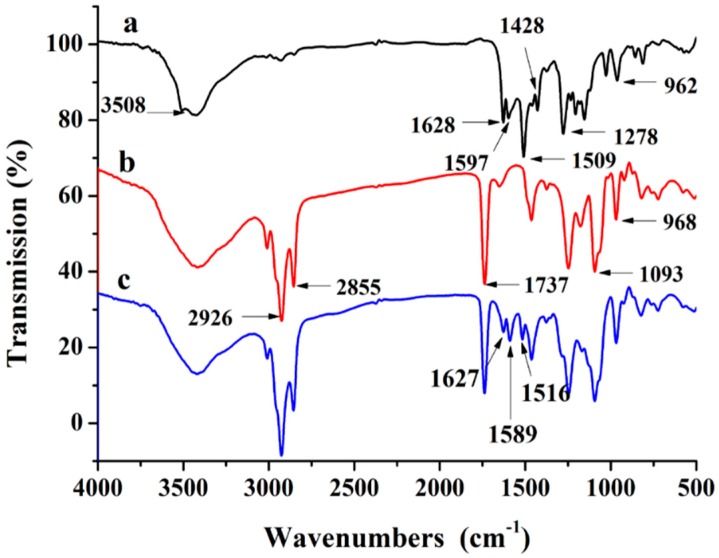
FT-IR spectrum of curcumin powder (**a**); phospholipid (**b**); and curcumin nanoliposome (**c**).

FT-IR was adopted to further verify whether curcumin was successfully incorporated into the nanoliposomes. The FT-IR spectrum of curcumin powder, phospholipid and curcumin nanoliposomes are shown in Figure 3a–c, respectively.

Curcumin showed its signature peaks at 3508 cm^−1^ (phenolic O-H stretching vibration), 1628 cm^−1^ (aromatic moiety C=C stretching), 1597 cm^−1^ (benzene ring stretching vibrations), 1509 cm^−1^ (C=O and C=C vibrations), 1428 cm^−1^ (olefinic C-H bending vibrations), 1278 cm^−1^ (aromatic C–O stretching vibrations), 1024 cm^−1^ (C–O–C stretching vibrations) [25]. The phospholipids displayed their typical peaks at 2926 and 2855 cm^−1^ (the CH_2_ stretching vibration), 1737 cm^−1^ (symmetrical C=O stretching vibration), 1648 cm^−1^ (water scissoring band) and 1249 cm^−1^ (PO_4_ antisymmetric stretching bands) [24]. When curcumin was incorporated in the nanoliposomes, the peak location and shape were similar to that of the phospholipids at 2926 cm^−1^ and 2855 cm^−1^ (CH_2_ vibration absorption), 1737 cm^−1^ (symmetrical C=O stretching vibration absorption) and 1249 cm^−1^ (PO_4_ antisymmetric stretching bands). Meanwhile, curcumin nanoliposomes exhibited peaks at 1627 cm^−1^ (aromatic moiety C=C stretching), 1589 cm^−1^ (benzene ring stretching vibrations) and 1516 cm^−1^ (C=O and C=C vibrations), which are the characteristic peaks of curcumin, indicating the existence of curcumin in the nanoliposomes. According to the viewpoint of Paramera *et al.* [26], the peaks of curcumin encapsulated in nanoliposomes were shifted from 1597 to 1589 and 1509 to 1516 cm^−1^, respectively, which indicated an interaction between curcumin and the phospholipids; the disappearance of the 3508 cm^−1^ peak in the curcumin nanoliposome spectrum indicated the interaction of the phenolic –OH of curcumin with phospholipid, most likely through hydrogen bonding. Curcumin’s main absorption peaks weakened and the peaks shifted when encapsulated in nanoliposomes, suggesting that the curcumin molecules were located inside the nanoliposomes, thus their spectrum “signature” was “hidden” [26].

### 2.2. In Vitro Drug Release of Curcumin Nanoliposome

**Figure 4:**
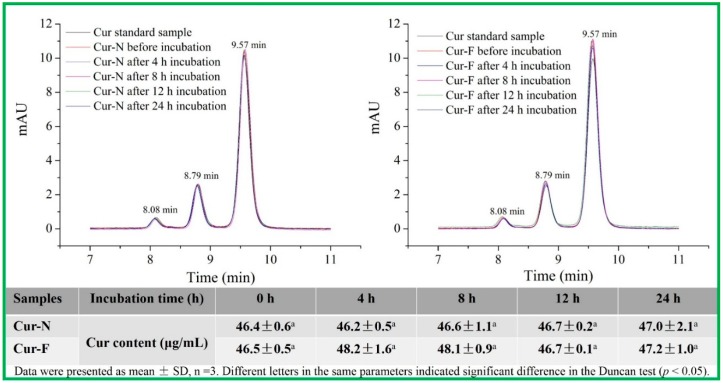
Structural integrity of curcumin under *in vitro* release conditions, Cur, Cur-F and Cur-N represent curcumin, free curcumin and curcumin nanoliposomes, respectively.

The integrity of curcumin under *in vitro* release conditions was analyzed using a HPLC before the sustained release experiments. As shown in Figure 4, the peaks of a curcumin standard sample appeared at 8.08 min, 8.79 min and 9.57 min, which were the retention times of bisdemethoxycurcumin, demethoxycurcumin and curcumin, separately, and this result was similar to the report of Syed *et al.* [27]. The retention times of the three forms of curcumin in both free curcumin and curcumin nanoliposomes under *in vitro* release conditions for 24 h were the same as those of the curcumin standard sample, and no significant change in the total curcumin content was observed, which indicated that curcumin possessed high stability and an integral structure under the *in vitro* release conditions.

**Figure 5:**
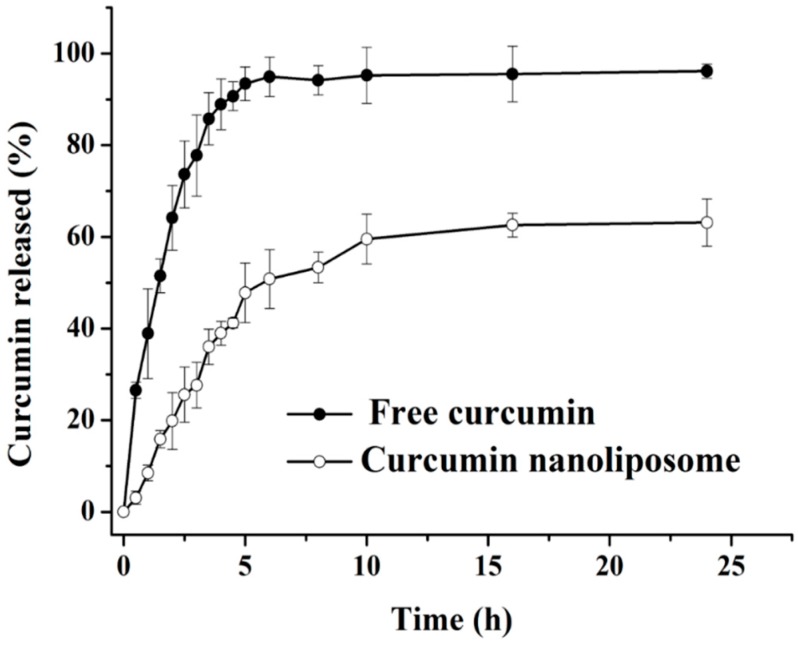
*In vitro* release of curcumin from free curcumin and curcumin nanoliposomes.

The sustained release properties of the curcumin nanoliposomes were further explored and are shown in Figure 5, taking free curcumin as a control group. Free curcumin displayed a fast diffusion rate across the dialysis bag, and approximately 93.4% ± 3.7% of curcumin diffused after 5 h. This was consistent with the results of Sun *et al.* [28] whereby nearly 91% of free curcumin diffused across the dialysis membrane after 6 h. However, curcumin nanoliposomes exhibited good sustained release. Only 19.8% ± 6.2%, 47.8% ± 6.5% and 63.1% ± 5.1% of curcumin was released from the nanoliposomes at 2, 5 and 24 h, respectively. The sustained release properties of curcumin in nanoliposomes was much better than that of poly (n-butylcyanoacrylate) nanoparticles [28], liposomes [14] and chitosan-coated liposomes [14], but poorer than BSA nanoparticles [29]. About 75% of curcumin loaded in poly(*n*-butyl cyanoacrylate) nanoparticles was released within 24 h [28]. Around 70% of curcumin was released from nanoparticles after 72 h, which was mainly attributed to that curcumin molecules loaded inside nanoparticles were not released, and curcumin molecules attached to the surface of nanoparticle could easily be released [29]. Liposomes and chitosan coated liposomes released 61.06% and 40.90% of curcumin after 6 h at 23 °C, respectively [14]. In the present work, the release rate of curcumin in nanoliposomes was much lower than that of free curcumin, most probably due to the fact curcumin was incorporated inside the nanoliposome bilayers, which could supress a burst release and retard its release rate.

### 2.3. Stability Studies of Curcumin Nanoliposomes

Curcumin is easily and rapidly degraded under neutral or basic pH conditions [16,30,31]. It could also easily chelate several metal ions in foods, beverages or even the blood of human body, thereby resulting in low pharmacological activities and instability [32]. Additionally, storage stability is an important feature of any curcumin delivery system. Consequently, whether nanoliposome encapsulation could improve the stability of the curcumin and the variation of curcumin nanoliposome stability during storage were explored in details in the present work.

#### 2.3.1. Stability against pH of Curcumin Nanoliposomes

In present work, the effect of nanoliposome encapsulation on preventing curcumin hydrolysis at a series of pH values was measured. As shown in Table 1, both free curcumin and curcumin nanoliposomes exhibited good stability at pH 6.5, with 98.7% ± 0.7% and 98.7% ± 2.8% of residual curcumin being observed in free curcumin and curcumin nanoliposomes after 3 h of incubation at pH 6.5. However, free curcumin was less stable than curcumin encapsulated in nanoliposomes at alkaline pHs. A fast degradation rate was observed in free curcumin after incubation at pH 7.4, 8.0, 10.0 and 12.0, as nearly 84.0% ± 0.8%, 80.1% ± 0.9%, 48.9% ± 5.0% and 24.8% ± 8.0% of curcumin decomposed in 40 min at pH 7.4, 8.0, 10.0 and 12.0. After 3 h incubation, there was only 12.1% ± 1.2%, 15.3% ± 0.7%, 17.2% ± 2.3% and 37.1% ± 1.9% curcumin remaining in free curcumin. The degradation rates of curcumin at pH 7.4 and 8.0 were much greater than that at pH 10.0 and 12.0. For example, nearly 78.2% ± 4.4% and 78.6% ± 1.2% of the curcumin degraded in 20 min at pH 7.4 and 8.0, and only 25.7% ± 4.9% and 15.2% ± 3.7% degradation of curcumin occurred at pH 10.0 and 12.0. It could be concluded from above results that curcumin was stable in acid solution and easily degraded in an alkaline environment; the degradation rate was significantly reduced with increasing pH. Wang *et al.* [17] also found the decomposition of curcumin was pH-dependent and occurred faster at neutral-basic conditions. After nanoliposome encapsulation, the pH stability of curcumin was significantly improved. Compared with free curcumin, curcumin nanoliposomes exhibited good stability against alkaline pHs (pH = 7.4, 8.0 and 10.0). Only 2.3% ± 2.7%, 3.2% ± 4.3% and 35.1% ± 10.1% degradation of curcumin in nanoliposomes was detected after 40 min incubation at pH 7.4, 8.0 and 10.0, respectively. There were still about 99.03% ± 7.5%, 90.8% ± 10.6%, and 40.1% ± 10.0% of residual curcumin in nanoliposomes after 3 h incubation at pH 7.4, 8.0, and 10.0, respectively. Other researchers also discovered curcumin located in glycyrrhetic acid-modified pullulan nanoparticles showed no significant degradation within 12 h under acidic neutral (pH 7.4) conditions [30].

**Table 1:** The stability of curcumin solution and curcumin nanoliposomes at pH 6.5, 7.4, 8.0, 10.0 and 12.0.

	pH	Samples	0 min	20 min	40 min	60 min	120 min	180 min
Curcumin residual rate	6.5	Cur-F	101.3 ± 2.7 ^a^	97.2 ± 5.4 ^a^	98.7 ± 5.6 ^a^	99.3 ± 2.1 ^a^	97.7 ± 3.2 ^a^	98.7 ± 0.7 ^a^
		Cur-N	99.5 ± 0.6 ^a^	99.1 ± 1.5 ^a^	99.5 ± 5.7 ^a^	99.3 ± 2.0 ^a^	99.6 ± 4.6 ^a^	98.7 ± 2.8 ^a^
	7.4	Cur-F	99.6 ± 3.6 ^a^	21.8 ± 4.4 ^b^	16.0 ± 0.8 ^b,c^	15.2 ± 1.2 ^b,c^	14.0 ± 1.5 ^b,c^	12.1 ± 1.2 ^c^
		Cur-N	99.3 ± 7.5 ^a^	94.2 ± 2.7 ^a^	97.7 ± 2.7 ^a^	100.5 ± 8.1 ^a^	97.7 ± 4.9 ^a^	99.3 ± 7.5 ^a^
	8.0	Cur-F	102.5 ± 9.8 ^a^	21.4 ± 2.0 ^c^	19.9 ± 0.9 ^c^	17.6 ± 0.7 ^c^	16.9 ± 1.9 ^c^	15.3 ± 0.7 ^c^
		Cur-N	100.1 ± 7.1 ^a,b^	96.9 ± 5.8 ^a,b^	96.8 ± 4.3 ^a,b^	94.4 ± 7.0 ^a,b^	89.7 ± 9.9 ^b^	90.8 ± 10.6 ^a,b^
	10.0	Cur-F	99.9 ± 9.0 ^a^	74.3 ± 4.9 ^b,c^	51.1 ± 5.0 ^e,f^	37.7 ± 6.5 ^g^	19.2 ± 2.4 ^h^	17.2 ± 2.3 ^h^
		Cur-N	96.8 ± 6.0 ^a^	80.5 ± 8.2 ^b^	64.9 ± 10.1 ^c,d^	64.0 ± 10.0 ^c,d^	54.9 ± 4.2 ^e,d^	40.1 ± 10.0 ^f,g^
	12.0	Cur-F	97.2 ± 18.4 ^a,b^	84.8 ± 3.7 ^b,c^	75.2 ± 7.9 ^c,d^	65.2 ± 7.0 ^d^	47.1 ± 5.7 ^e^	37.1 ± 1.9 ^e,f^
		Cur-N	99.3 ± 7.1 ^a^	81.9 ± 9.3 ^c^	67.7 ± 2.2 ^d^	46.0 ± 2.5 ^e^	32.5 ± 5.0 ^f,g^	22.2 ± 4.1 ^g^

Data were presented as mean ± SD, *n* = 3. Different letters in the same parameters indicate a significant difference in the Duncan test (*p* ˂ 0.05). Cur-F and Cur-N represent free curcumin and curcumin nanoliposomes, respectively.

It could be concluded from the above results that nanoliposome encapsulation could protect curcumin from degradation in alkaline environments. Curcumin is thought to adopt a trans-bilayer orientation in the phospholipid bilayer of liposomes [33]. That is, one phenoxy group resides near the membrane/water interface, while the keto-enol group and the other phenoxy group are buried within the hydrophobic core of the liposome [33]. The keto–enol group was effectively protected when the curcumin molecule was incorporated inside the phospholipid bilayer of the liposome. In addition, the hydrogen bond interaction between the phenoxy group of curcumin and the phospholipid headgroup also plays and important role in preventing curcumin degradation [6].

Curcumin encapsulated in nanoliposomes became less stable at pH = 12.0, and 22.2% ± 4.1% of the curcumin remained in the nanoliposomes after 3 h incubation at this pH, while 37.1% ± 1.9% of curcumin was retained in free curcumin. This indicated that nanoliposome encapsulation could promote the degradation of curcumin at high alkaline pH values. This was consistent with the results of Leung, *et al.* [34] that curcumin was dissociated from micelles into the aqueous phase at high alkaline pH (pH = 13). The absence of encapsulation and stabilization by the micellar solution resulted in rapid hydrolysis of curcumin [34]. In the present work, high alkalinity might cause the degradation of the liposome phospholipid layer, resulting in the rapid leakage and hydrolysis of curcumin.

#### 2.3.2. Stability against Metal Ions of Curcumin Nanoliposomes

Several functional groups of curcumin including the carbonyl and enolic groups of the β-diketone moiety, α,β-unsaturated β-diketone moiety and phenyl rings were found to apt at interacting with other molecules [35]. The presence of metal ions can also result in the instability of curcumin. The chelation activity of curcumin could reduce its stability and pharmacological activities.

**Figure 6:**
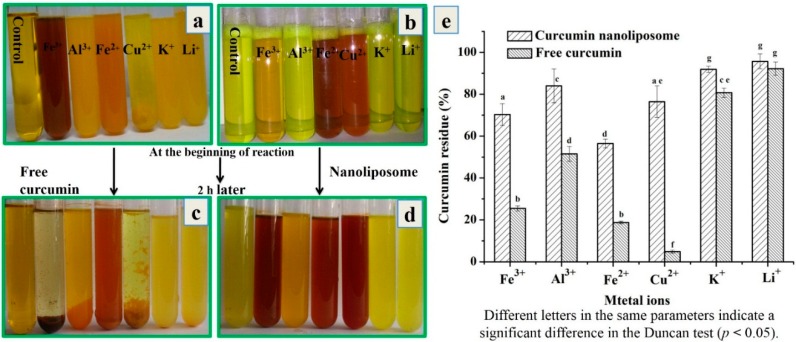
Photographs of mixtures of free curcumin-metal ion solutions (**a**); curcumin nanoliposomes-metal ion solutions (**b**) at the beginning of reaction; free curcumin-metal ion solutions (**c**); curcumin nanoliposomes-metal ion solutions (**d**) after 2 h of incubation; (**e**) residual curcumin amount in the mixture of free curcumin-metal ion solutions (**c**); curcumin nanoliposomes-metal ion solutions after 2 h of incubation.

In this study, we inspected whether nanoliposome encapsulation could improve the stability of curcumin against metal ions. As shown in Figure 6, free curcumin could chelate with metal ions (Fe^3+^, Al^3+^, Fe^2+^ and Cu^2+^), and sediments with different colors were generated. There were no precipitation and no color change after curcumin was mixed with K^+^ or Li^+^ ions. The remaining curcumin in mixtures of Fe^3+^, Al^3+^, Fe^2+^ or Cu^2+^ ions and free curcumin was 25.5% ± 1.2%, 51.5% ± 3.6%, 18.8% ± 0.7% and 4.8% ± 0.7%, which indicated that the most of curcumin chelated with these metal ions. Among these metal ions, curcumin chelated with Cu^2+^ the fastest, and the sediment was generated immediately after free curcumin was poured into Cu^2+^ solution. Meanwhile 80.7% ± 2.2% and 92.2% ± 3.2% of curcumin still remained in the mixtures of K^+^ or Li^+^ ions and free curcumin, indicating few interactions occurred between curcumin and these ions. This was consistent with the results of Baum and Ng [36] that Cu^2+^ or Fe^2+^ ion could each bind at least two curcumin molecules.

After encapsulation in nanoliposomes, the stability of curcumin against metal ions was improved. There was no sediment in curcumin nanoliposomes after mixing with metal ions. The color of curcumin nanoliposomes mixed with Fe^3+^, Fe^2+^ and Cu^2+^ ions changed at the beginning of the reaction, but the color of curcumin nanoliposomes with Al^3+^, K^+^ and Li^+^ ions changed after a period of time. After 2 h of incubation, the remaining curcumin in nanoliposomes was much higher than that in free curcumin solution, and there was 70.3% ± 5.2%, 84.0% ± 8.1%, 56.5% ± 2.1%, 76.5% ± 7.5%, 91.8% ± 1.5% and 95.7% ± 3.5% of curcumin remaining in Fe^3+^, Al^3+^, Fe^2+^, Cu^2+^, K^+^ and Li^+^ ions solution.

The high stability of curcumin after encapsulation was attributed to the high stability of curcumin in nanoliposomes. The active keto-enol group and the phenoxy group were buried within the hydrophobic core of the nanoliposomes, and the deep penetration of the reactive keto-enol group inside the phospholipid bilayer could lead to high stability of nanoliposome curcumin [6]. Meanwhile, the nanoliposome membrane could also prevent the inner curcumin from directly interacting with metal ions, and avoid the chelation reaction between curcumin and metal ions.

#### 2.3.3. Storage Stability of Curcumin Nanoliposomes

**Figure 7:**
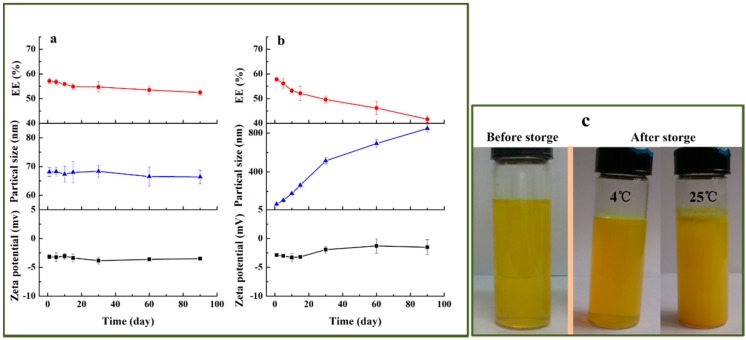
The changes in encapsulation efficiency, average diameter, zeta potential and of curcumin nanoliposomes during storage at 4 (**a**) and 25 °C (**b**); the image before and after 90 days of storage; (**c**) photographs of curcumin nanoliposomes stored at 4 and 25 °C.

Storage stability is an important index of any drug delivery system. Physical instability would lead to drug leakage and aggregation or fusion of vesicles [37]. In this work, the physical stability of curcumin nanoliposomes including encapsulation efficiency, average diameter and zeta potential was evaluated. Curcumin nanoliposomes were separately stored at 4 and 25 °C for 90 days. The physical stability results are shown in Figure 7. When stored at 4 °C, the size and zeta potential of curcumin nanoliposomes did not change obviously, and the encapsulation efficiency of curcumin nanoliposomes displayed a slight decrease after 90 days of storage. However, when stored at 25 °C, the zeta potential only changed from −2.88 ± 0.22 mV to −1.49 ± 1.31 mV, but the average diameter began to increase rapidly, and grew from 68.1 ± 1.5 to 847.9 ± 38.7 nm after 90 days of storage. The encapsulation efficiency decreased from 57.8% ± 0.7 % to 41.7% ± 1.4% after 90 days of storage. This indicated that curcumin nanoliposomes had favorable physical stability when stored at 4 °C, which was attributed to the fact that the low temperature inhibited the decomposition of the nanoliposomes [29].

### 2.4. Cellular Antioxidant Activity of Curcumin Nanoliposomes

Curcumin has been found to be an effective and safe natural antioxidant in different *in vitro* assays [38]. Liposomal curcumin exhibited higher stability and DPPH scavenging activity than free curcumin, which is based on a simple chemical reaction [6]. Biological systems are much more complex and it is unscientific to predict the biological activity of curcumin nanoliposomes *in vivo* simply by traditional chemical antioxidant assays. It is important to adopt CAA to evaluate the antioxidant ability of curcumin before and after nanoliposome encapsulation.

**Figure 8:**
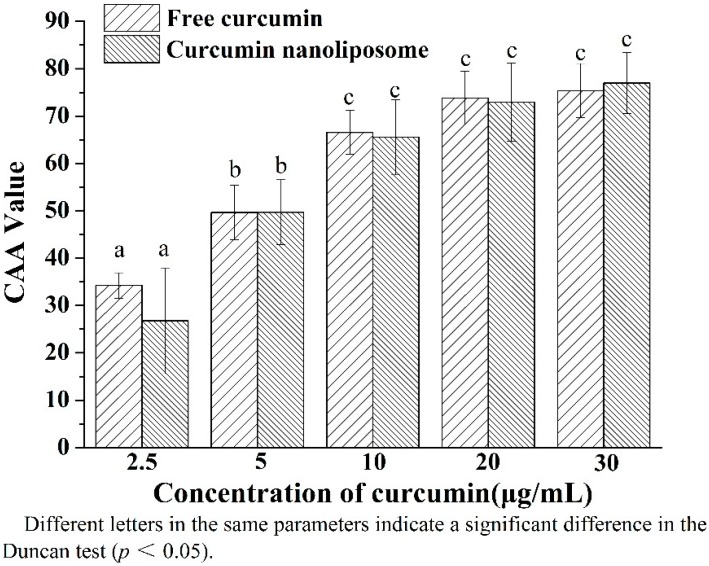
Celluar antioxidant activity of free curcumin and curcumin nanoliposomes.

As shown in Figure 8, curcumin nanoliposomes exhibited comparable CAA with free curcumin at the same concentration. The CAA value of curcumin nanoliposomes and free curcumin increased with the increasing concentration of curcumin. The encapsulation of curcuminoids in modified ε-polylysine-enhanced CAA of curcuminoids, which was mainly attributed to higher solubility and lower degradation of curcuminoids after encapsulation, and specific interaction with the HepG2 cells [31]. In the present work, the equal CAA value of curcumin before and after encapsulation in nanoliposome might be attributed to the synthetical effect of curcumin protective capability, sustained released and cellular uptake of curcumin nanoliposomes. The curcumin protective capability of nanoliposomes in a slightly alkaline environment could increase the antioxidant activity of curcumin. However, the sustained release properties of curcumin nanoliposome would decrease the effective concentration of curcumin. Additionally, low cellular uptake of curcumin nanoliposome would result in a low concentration of curcumin in the cell and low CAA, which will be further measured in the following text.

### 2.5. Cellular Uptake Assays of Curcumin Nanoliposomes

**Figure 9:**
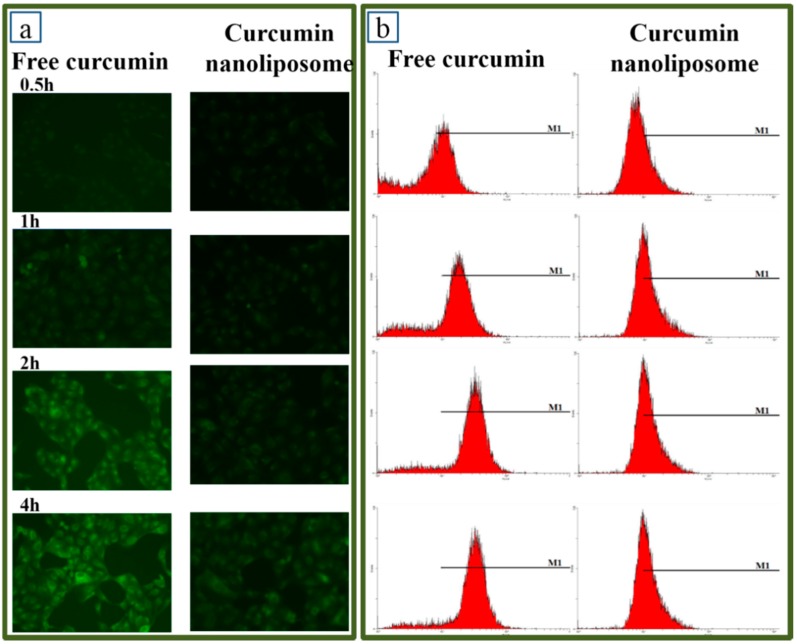

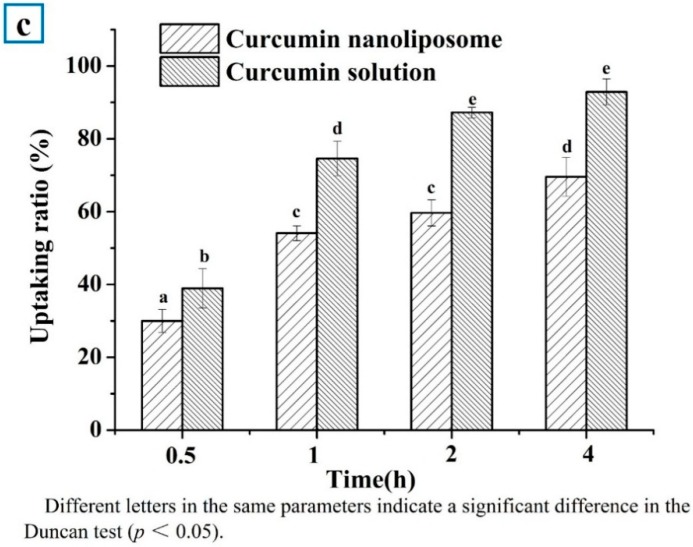
Fluorescence microscope analysis of Caco-2 cells treated with 20 μg/mL free curcumin and curcumin nanoliposome after a defined period of time (**a**); Flow-cytometric analysis of Caco-2 cells treated with 20 μg/mL free curcumin and curcumin nanoliposome after a defined period of time (**b**); Uptaking ratio analysis of Caco-2 cells treated with 20 μg/mL free curcumin and curcumin nanoliposome after a defined period of time (**c**).

Cellular uptake assays were investigated to further explain the equal CAA value of curcumin before and after encapsulation in nanoliposomes. Curcumin possesses an inherent green fluorescence [39], which could be utilized to measure the cellular uptake profiles of curcumin before and after encapsulation in nanoliposomes. The cellular uptakes of curcumin nanoliposomes and free curcumin were determined by fluorescence microscopy at different time intervals, giving the results shown in Figure 9a. The fluorescence intensity of Caco-2 cells treated with free curcumin or curcumin nanoliposomes increased with the extension of incubation time by 2 h. The fluorescence intensity reached saturation and did not increase after 2 h of incubation. Additionally, fluorescence microscopy showed that Caco-2 cells treated with free curcumin exhibited higher fluorescence intensity than the cells treated with curcumin nanoliposomes. It indicated that free curcumin possessed higher cellular uptake ratio than curcumin nanoliposomes. Flow cytometry was further used to quantify the difference in cellular uptake of curcumin before and after nanoliposome encapsulation. The flow cytometry results of free curcumin and curcumin nanoliposomes are shown in Figure 9b,c. The cellular uptake rates were 38.9% ± 5.4%, 74.6% ± 4.7%, 87.2% ± 1.5% and 92.9% ± 3.6% for free curcumin and 29.9% ± 3.2%, 54.1% ± 2.0%, 59.7% ± 3.6% and 69.6% ± 5.4% for curcumin nanoliposomes after 0.5, 1, 2 and 4 h of incubation, respectively. The relative fluorescence values indicated the cellular uptake of curcumin was lowered after encapsulation in nanoliposomes, which was consistent with the fluorescence microscopy analysis results.

The free curcumin solution used in the present experiments was dissolved in DMSO before dilution. Free curcumin (lipid-soluble drug) can be delivered into cells passively through diffusion. However, liposomes are transferred to the cells via clathrin-mediated endocytosis [40]. The transfer of drugs encapsulated in nanocarriers with good sustained release property into cells mainly relies on the contribution of endocytosis, and the endocytosis pathway has lower power to deliver the drug into cells than the passive diffusion pathway [41].

A lower curcumin nanoliposome delivery power led to its lower cellular uptake rate. Additionally, the lower cellular uptake rate of curcumin nanoliposomes caused by the good sustained release properties seemingly trended to result in lower CAA of curcumin nanoliposomes in comparison with free curcumin. As a matter of fact, nanoliposomes provide shelter for intracellular curcumin, and free curcumin exhibited less stability against the intracellular environment. This would contribute to the equal CAA value of curcumin before and after encapsulation in nanoliposomes.

## 3. Experimental Section

### 3.1. Materials

Curcumin (98%) and 2,2′-azobis (2-amidinopropane) dihydrochloride (ABAP) were purchased from Aladdin Industrial Corporation (Shanghai, China). Phospholipid S100 was provided by Lipoid GmbH (Ludwigshafen, Germany). Cholesterol and 2′,7′-dichlorofluorescin diacetate (DCFH-DA) were obtained from Sigma-Aldrich Chemical Co. (St. Louis, MO, USA). Dulbecco’s modification of Eagle’s medium was purchased from Beijing Solarbio Science & Technology Co., Ltd. (Beijing, China). Fetal bovine serum was obtained from Shanghai ExCell Biology. Inc. (Shanghai, China). Methylthiazolyl tetrazolium (MTT), ethanol, Tween 80, methanol, and other reagent chemicals were all of analytical grade.

### 3.2. Curcumin Nanoliposome Preparation

The curcumin nanoliposomes were prepared using the thin-film dispersion method combined with dynamic high-pressure microfluidization (DHPM) according to our previous study [22,23]. Firstly, phospholipid, cholesterol, Tween-80 and curcumin at a mass ratio of 28:4.3:7.8:1 were fully dissolved in ethanol, and then transferred into a round bottom flask. The ethanol was evaporated on a rotary evaporator under vacuum at 55 °C to form a thin film. The dried lipid film was further rehydrated with phosphate buffer solution (pH 6.5) to form a coarse liposome suspension. Secondly, the coarse liposome suspension was exposed to a microfluidizer (M-110EH30, Microfluidic Corp., Newton, MA, USA) at a pressure of 120 MPa for three cycles. Curcumin nanoliposomes with a final phospholipid concentration of 14 mg/mL were thus obtained. 

### 3.3. The Solubility of Curcumin in Nanoliposomes

In order to determine the solubility of curcumin in nanoliposomes, curcumin nanoliposomes were centrifuged at 1500 g for 15 min to remove any curcumin crystals, then aliquots of supernatant were collected for UV spectrophotometry analysis.

### 3.4. Physicochemical Characterization and Morphology of Curcumin Nanoliposomes

The encapsulation efficiency of curcumin nanoliposome was measured by separating non-encapsulated curcumin through exclusion chromatography, which was done according to the method of Pellequer *et al.* [42]. The gel was first saturated with blank nanoliposomes. An aliquot of 1 mL curcumin-loaded nanoliposomes was added and eluted at a flow-rate of 1 mL/min using deionized water as the mobile phase. The filtrate was collected and diluted with methanol, then the curcumin content in the filtrate was detected by UV spectrophotometry. The entrapment efficiency (EE) of curcumin nanoliposome was calculated as:

EE% = (*W_en_*/*W_total_*) × 100% (1)

where *W_En_* was the amount of curcumin incorporated into the nanoliposomes, and *W_total_* was initial weight of curcumin added during the preparation. 

The size distribution and zeta potential values of curcumin nanoliposome were measured according to our previous method [24] using a dynamic laser light scattering (DLS) instrument (Nicomp 380 ZLS, Santa Barbara, CA, USA) with the intensity at an angle of 90° at 25 °C. Nanoliposomes were diluted 10-fold with Milli-Q water before analysis. An individual zeta potential value was calculated from the average of at least 10 readings of each sample. The mean particle size and zeta potential were calculated as the average value of triplicate measurements. 

The morphology of nanoliposomes was analyzed through TEM according to our previous research [24]. A drop of curcumin nanoliposome suspension which was diluted to a final phospholipid concentration of 1 mg/mL with distilled water was placed onto a carbon coated copper grid. After 4 min, the copper mesh grid was stained with phosphotungstic (1%) for another 4 min. The excess liquid was removed with filter paper. The sample was then air-dried at room temperature and observed under a TEM (JEM-2100, JEOL, Tokyo, Japan) at a voltage of 200 kV.

The infrared spectra of curcumin nanoliposome, phospholipid and curcumin were separately obtained according to our previous study [43]. An appropriate amount of KBr was separately mixed with curcumin nanoliposomes, phospholipid and curcumin, ground and formed into a disk. The disk was scanned by an infrared spectrometer over the range from 4000 to 400 cm^−1^.

### 3.5. In Vitro Drug Release of Curcumin Nanoliposomes

The stability and integrity of curcumin under *in vitro* release conditions was analyzed before the *in vitro* drug release experiments. Aliquots of free curcumin and curcumin nanoliposomes were mixed with the receptor medium used in the drug release experiment, respectively, and the mixtures were incubated at 37 °C for 24 h. At defined times, samples were taken for HPLC analysis (Agilent 1260 series, Santa Clara, CA, USA), using a Sunfire C18 column (5 μm, 250 mm × 4.6 mm; Waters, Milford, MA, USA) connected to a UV detector (Agilent 1260 VWD VL). According to the method described by Chen, *et al.* [13] with slight modification, the mobile phase consisted of 1% glacial acetic acid and acetonitrile at a ratio of 45/55 (*v*/*v*) and a flow rate of 1.0 mL/min at 25 °C, and chromatograms were recorded at 427 nm. Total curcumin content was calculated according to a standard curve equation (*y* = 74.844*x* + 2.5383, *R*^2^ = 0.9998). *In vitro* drug release of curcumin nanoliposomes was measured according to the method of Chen *et al.* [13]. An amount of nanoliposomes or free curcumin solution (2 mL) were separately loaded into a dialysis bag with a cutoff size of 10 kDa which was placed in 100 mL of receptor medium (kept constant at 37 ± 0.5 °C and stirred at 100 rpm). The composition of the receptor medium was 0.5% Tween-80 and 25% (*v*/*v*) ethanol dissolved in PBS (pH 6.5, 0.01 M). The content of curcumin in the samples was determined at predetermined time intervals using UV spectrophotometry and calculated according to a calibration curve.

### 3.6. Curcumin Nanoliposome Stability Studies 

#### 3.6.1. pH Stability of Curcumin Nanoliposomes

Several researchers have shown that the decomposition of curcumin was pH-dependent and the degradation of curcumin was rapid under neutral or basic conditions [44]. There is a report that about 90% of curcumin decomposed within 30 min when incubated in phosphate buffer (pH 7.2) and the stability of curcumin was strongly improved by lowering the pH [17]. Therefore, various pH conditions (pH = 6.5, 7.4, 8.0, 10.0 and 12.0) were selected to evaluate whether nanoliposomes could increase the stability of curcumin after exposure to alkaline pHs, by taking free curcumin as a control group.

An appreciable amount of curcumin nanoliposomes and free curcumin was added into a series of pH solutions (pH = 6.5, 7.4, 8.0, 10.0 and 12.0) with the ratio of 1:10. All samples were incubated in a water bath at 37 °C with a stirring rate of 100 rpm. Sample aliquots were collected at predetermined time intervals (0, 20, 40, 60, 120 and 180 min) and then the residual amount of curcumin was determined using UV spectrophotometry at 425 nm. 

#### 3.6.2. Stability of Curcumin Nanoliposomes against Metal Ions

The stability of curcumin nanoliposomes against metal ions(Fe^3+^, Al^3+^, Fe^2+^, Cu^2+^, K^+^, Li^+^) was determined out according to the method of Zhao, *et al.* [18] with a few modifications, by taking free curcumin as a control group. An appropriate amount of FeCl_3_, AlCl_3_, FeCl_2_, CuCl_2_, KCl and LiCl were weighed separately to prepare 1 mmol/L metal ions solutions. Curcumin nanoliposomes or free curcumin (2 mL) were added separately to the test tube, and mixed with different metal ions solution and then incubated in a water bath at 37 °C for 2 h. The reactants were centrifuged at 8000 rpm for 20 min, aiming to separate the curcumin metal chelate sediments from the supernatant. The supernatant was diluted with methanol, and curcumin content determined using UV spectrophotometry at 425 nm and calculated according to a calibration curve.

#### 3.6.3. Storage Stability of Curcumin Nanoliposomes

Curcumin nanoliposomes were stored at 4 °C or 25 °C for 3 months in sealed containers in the dark. Variation in the encapsulation efficiency, particle size and zeta potential were then determined at predetermined time intervals (1, 5, 10, 15, 30, 60 and 90 days)

### 3.7. Cellular Antioxidant Activity of Curcumin Nanoliposomes

Cellular antioxidant activity was measured according to the method of Wolfe, *et al.* [45] and Hu, *et al.* [46] with a few modifications. Caco-2 at a density of 5 × 10^4^ cells was seeded in a black 96-well plate (200 μL/well). After 24 h of incubation, the medium was removed and the wells were washed with PBS. Then the cells were treated for 1 h with free curcumin or curcumin nanoliposomes in different concentrations and 25 µmol/L DCFH-DA dissolved in treatment medium. After 1 h of incubation, drugs and fluorescent probe was removed and the plate was washed with PBS. The plate was refilled with 600 µmol/L ABAP dissolved in 100 μL PBS. Fluorescence of cells in 96-well microplate within 1 h was read every 5 min at 37 °C. The emission and excitation wavelengths were 538 nm and 485 nm, respectively. The control group was treated with DCFH-DA and ABAP, and blank group was only treated with DCFH-DA. Other processing were all the same.

### 3.8. Cellular Uptake Assays of Curcumin Nanoliposomes

The intracellular antioxidant activity of curcumin was related with the cellular uptake of curcumin. A comparative visualization of free curcumin or curcumin nanoliposomes was carried out to evaluate their uptake in Caco-2 cells. Caco-2 cells (1 mL) were seeded into 12-well plates. After 24 h of incubation, the medium was replaced with fresh medium containing 20 μg/mL of curcumin nanoliposomes or free curcumin. The cells were washed with PBS and examined under an inverted fluorescence microscope at different time intervals (0.5, 1, 2 and 4 h). To further quantify the difference in cellular uptake of free curcumin and curcumin nanoliposomes, Caco-2 cells were seeded into 12-well plates and treated with these samples at predetermined time intervals. After incubation, the medium was removed and washed with PBS. Then cells were trypsinized, centrifuged and collected in PBS. The cell suspension was injected into an Accuri C6 flow cytometer (Accuri Cytometers, Inc., Ann Arbor, MI, USA) in the FL1 channel (488 excitation, blue laser, 530 ± 15 nm, FITC/GFP) to determine the fluorescence intensity [25].

### 3.9. Statistical Analysis

Data were reported as mean ± standard deviation (S.D.). The date were from at least three independent experiments. Statistical analysis was carried out using SPSS software version 17.0 (IBM, Hong Kong, China). Differences were considered significant at *p* < 0.05.

## 4. Conclusions

This study has systematically evaluated the physicochemical properties of curcumin nanoliposomes, including particle size distribution, zeta potential, morphology and sustained release. The stabilities of curcumin loaded in nanoliposomes against pH and metal ions was explored in detail. The stability of curcumin was significantly improved by encapsulating in nanoliposomes. Compared with free curcumin, curcumin nanoliposomes exhibited equal CAA. Curcumin loaded in nanoliposomes also exhibited lower curcumin cellular uptake than free curcumin, which was a vital factor for the low CAA of curcumin nanoliposomes. 
